# Control of Grain Shape and Size in Rice by Two Functional Alleles of *OsPUB3* in Varied Genetic Background

**DOI:** 10.3390/plants11192530

**Published:** 2022-09-27

**Authors:** Zhu-Hao Li, Shi-Lin Wang, Yu-Jun Zhu, Ye-Yang Fan, De-Run Huang, Ai-Ke Zhu, Jie-Yun Zhuang, Yan Liang, Zhen-Hua Zhang

**Affiliations:** 1 State Key Laboratory of Rice Biology and Chinese National Center for Rice Improvement, China National Rice Research Institute, Hangzhou 310006, China; 2 Nanchong Academy of Agricultural Sciences, Nanchong 637000, China

**Keywords:** gene cloning, grain shape and size, *OsPUB3*, QTL, rice

## Abstract

Grain shape and size are key determinants of grain appearance quality and yield in rice. In our previous study, a grain shape QTL, *qGS1-35.2*, was fine-mapped using near-isogenic lines (NILs) derived from a cross between Zhenshan 97 (ZS97) and Milyang 46 (MY46). One annotated gene, *OsPUB3*, was found to be the most likely candidate gene. Here, knockout and overexpression experiments were performed to investigate the effects of *OsPUB3* on grain shape and size. Four traits were tested, including grain length, grain width, grain weight, and the ratio of grain length to width. Knockout of *OsPUB3* in NIL^ZS97^, NIL^MY46^, and another rice cultivar carrying the *OsPUB3*^MY46^ allele all caused decreases in grain width and weight and increases in the ratio of grain length to width. Results also showed that the magnitude of the mutational effects varied depending on the target allele and the genetic background. Moreover, it was found that NIL^ZS97^ and NIL^MY46^ carried different functional alleles of *OsPUB3*, causing differences in grain shape rather than grain weight. In the overexpression experiment, significant differences between transgenic-positive and transgenic-negative plants were detected in all four traits. These results indicate that *OsPUB3* regulates grain shape and size through a complex mechanism and is a good target for deciphering the regulatory network of grain shape. This gene could be used to improve grain appearance quality through molecular breeding as well.

## 1. Introduction

Rice (*Oryza sativa* L.) provides a staple food source for more than half the world’s population. Grain shape and size are important appearance quality of rice, which are one of the most direct characteristics for consumers and influence the market value of grain products. In general, long and slender grains have higher competitive market value, which is preferred by consumers in most regions of the world [1,2,3,4]. Grain size is also a major determinant of grain weight, one of the three yield components (number of panicles per plant, number of grains per panicle, and grain weight). Therefore, understanding the genetic basis of grain shape and size is vital for improving grain quality and yield of rice.

Grain shape and size are largely determined by grain length and width, which are controlled by a large number of quantitative trait loci (QTL). At least 25 QTLs for grain shape and size in rice have been cloned. Fifteen of them mainly regulated grain length, including *qTGW1.2b* [5], *GS2*/*GL2* [6,7], *OsLG3* [8], *OsLG3b*/*qLGY3* [9,10], *GS3.1* [11], *GS3* [12], *SG3* [13], *GL3.1*/*qGL3* [14,15], *qTGW3* [16], *qGL5* [17], *TGW6* [18], *GW6a* [19], *GL6* [20], *GLW7* [21] and *GL10*/*OsMADS56* [22,23]. Seven other QTLs mainly regulated grain width, including *GW2* [24], *TGW2* [25], *GS5* [26], *GSE5* [27], *GW6* [28], *GW8* [29] and *GW10* [30]. The remaining three genes, including *GSA1* [31], *GL7*/*GW7* [32,33], and *GS9* [34], exhibited similar effects on grain length and width. All of these QTLs conferred significant influence on grain weight, except *GL7*/*GW7* and *GS9*. Characterization of these QTLs has greatly enriched our knowledge of the genetic control of grain size in rice. Some elite alleles of these QTLs have been tried for targeted improvement of rice by genome editing system [3,35,36,37]. However, much more effort is needed to fully understand the regulatory mechanisms of grain shape and size [4,38,39]. Moreover, the identification of more genes could provide the flexibility needed to design various rice grain shapes [4].

Ubiquitination is a post-translational modification that regulates protein stability. Ubiquitination requires a series of enzymes, including ubiquitin-activating enzyme (E1), ubiquitin-conjugating enzyme (E2), and ubiquitin-protein ligase (E3) [40,41,42]. E1 activates ubiquitin and transfers it to E2. E3 promotes the transfer of the ubiquitin conjugated by E2 to the substrate. In the ubiquitination process, the substrate is mainly recognized by E3 which is usually classified into three groups: the RING and U-box type, the HECT type, and the RBR type [43]. In addition, ubiquitination is dynamic and can be removed by deubiquitinating enzymes [43]. The ubiquitin-proteasome pathway is known to play an important role in regulating grain shape and size. *GW2* was the first cloned QTL for grain width and weight, which encodes a RING-type E3 ubiquitin ligase [24]. A recent study revealed that GW2 could ubiquitinate a glutaredoxin protein WG1 and direct WG1 to the proteasome for degradation. The degradation eliminated the repression of the transcriptional activity of OsbZIP47 by WG1 and promoted the transcription of downstream genes, which consequently regulated grain shape and weight [44]. Another gene, *WTG1*/*qNPT1* identified as a major QTL determining the “new plant type” architecture [45], encoded a deubiquitinating enzyme with homology to human OTUB1 and negatively regulated grain width and weight [46]. WTG1/qNPT1 could physically interact with the E2 ubiquitin-conjugating protein OsUBC13 and transcription factor OsSPL14. This interaction affected ubiquitination and proteasomal degradation of OsSPL14 [45].

A minor QTL for grain shape, *qGS1-35.2*, was previously fine-mapped into a 57.7-kb region on chromosome 1 using near-isogenic lines (NILs) derived from a cross between *indica* rice cultivars Zhenshan 97 (ZS97) and Milyang 46 (MY46). One annotated gene, *Os01g0823900* encoding a U-box type E3 ubiquitin ligase OsPUB3, was found to be the most likely candidate gene [47]. In order to investigate the effect of *OsPUB3* on grain shape and size, we performed knockout and overexpression experiments in the present study. Knockout of *OsPUB3* in three rice cultivars carrying the ZS97 or MY46 allele all caused changes in grain shape and size, and the magnitude of a mutational effect varied depending on the target allele and genetic background. Overexpression of *OsPUB3* also brought about changes in grain shape and size, whereas the effect of overexpression was not exactly opposite to the effect of knockout experiments. These results suggested that *OsPUB3* regulates grain development through a complex mechanism and is a good target gene for deciphering the regulatory network of grain shape and size in rice. In addition, *OsPUB3* may coordinate the trade-off between grain weight and other yield traits. Knockout reduced grain weight but increased panicle or grain number, resulting in stable grain yield. Therefore, *OsPUB3* could be used to improve grain appearance quality without yield penalty. Overall, our study provides a new genetic resource to improve grain appearance quality and explore the regulatory framework for grain shape and size.

## 2. Results

### 2.1. Knockout Mutants of OsPUB3 Produced from Three Rice Cultivars

**Figure 1:**
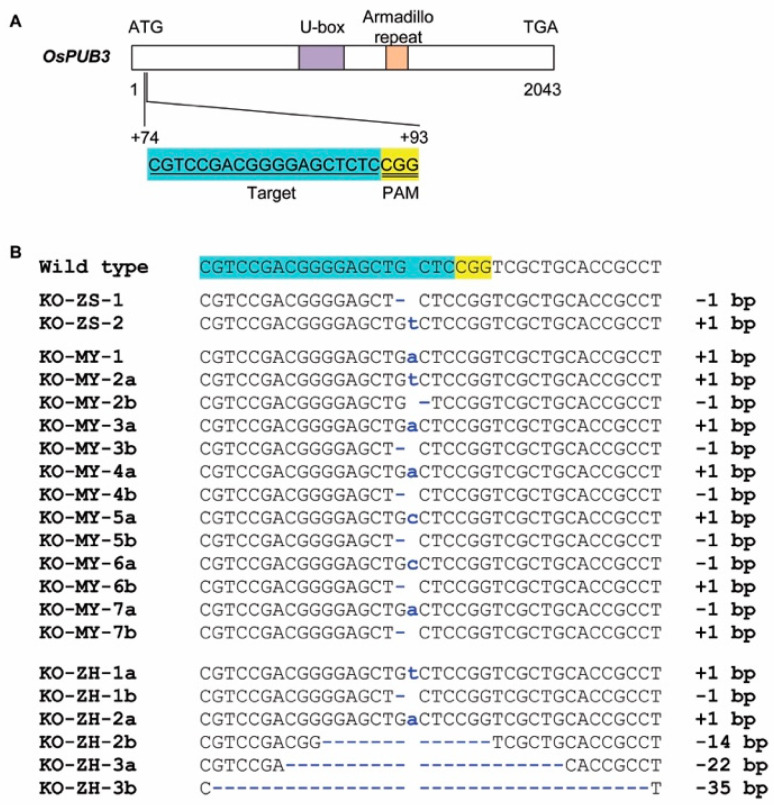
Knockout of *OsPUB3* in three rice cultivars. (**A**) The schematic of *OsPUB3* gene structure and the CRISPR/Cas9 target site. *OsPUB3* has only one exon indicated by black rectangles. The translation initiation codon (ATG) and termination codon (TGA) are shown. OsPUB3 protein contains a U-box motif and an Armadillo repeat. (**B**) Sequence mutations in the target region. Mutations are indicated by blue letters. KO-ZS-1 and KO-ZS-2 were two homozygous mutants in the NIL^ZS97^ background. KO-MY-1 was a homozygous mutant in the NIL^MY46^ background; KO-MY-2 to KO-MY-7 were six biallelic mutants in the NIL^MY46^ background. KO-ZH-1, KO-ZH-2 and KO-ZH-3 were three biallelic mutants in the ZH161 background.

**Figure 2:**
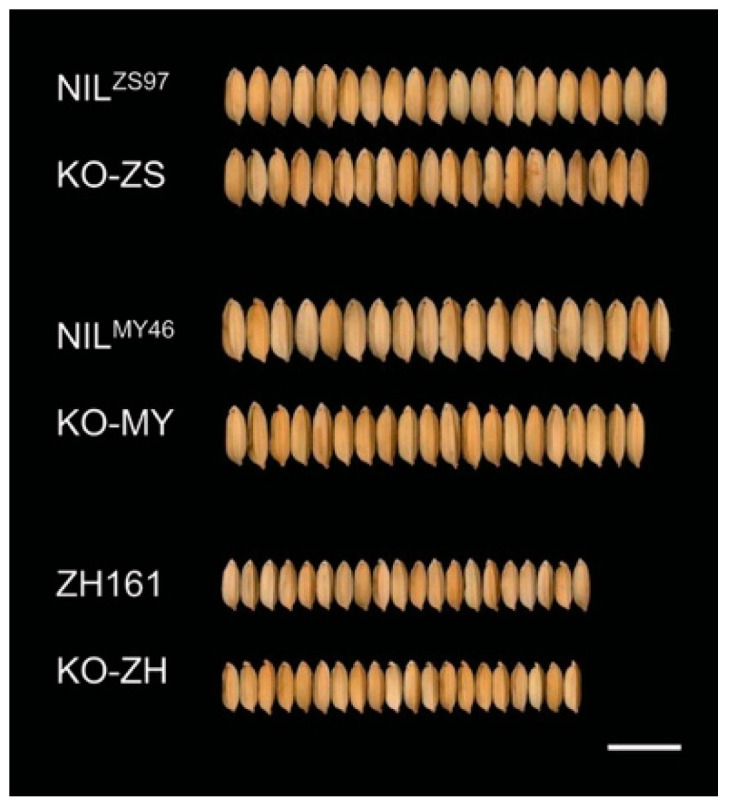
Grains of the recipients and knockout mutants. Bar = 10 mm.

The CRISPR/Cas9 system was used to produce mutants for validating the effects of *OsPUB3*. OsPUB3 protein contained a U-box motif and Armadillo (ARM) repeats in the central region. A site located in the distal N-terminal region was selected as the target for CRISPR/Cas9 gene editing (Figure 1A). To compare the effects of the two parental alleles, *OsPUB3^ZS97^* and *OsPUB3^MY46^*, and to test the effects of *OsPUB3* in different genetic backgrounds, three recipients were used. They are NIL^ZS97^ and NIL^MY46^ previously used for fine-mapping *qGS1-35.2*, and another *indica* rice cultivar carrying the *OsPUB3^MY46^* allele, Zhonghui 161 (ZH161). NIL^ZS97^ and NIL^MY46^ have a small difference in grain shape and size, whereas the grain of ZH161 is much thinner and smaller (Figure 2).

A total of twelve independent T_0_ mutants were obtained, including three homozygous and nine biallelic mutants (Figure 1B). For NIL^ZS97^, two homozygous mutants and one transgenic-negative control were identified. KO-ZS-1 had a 1 bp deletion and KO-ZS-2 had a 1 bp insertion. For NIL^MY46^, one homozygous and six biallelic mutants were identified. No negative transformant was found. The homozygous mutant had a 1 bp insertion, and each of the biallelic mutants contained a 1 bp insertion and a 1 bp deletion. For ZH161, three biallelic mutants and one negative transformant were identified. KO-ZH-1 contained a 1 bp insertion and a 1 bp deletion, KO-ZH-2 contained a 1 bp insertion and a 22 bp deletion, and KO-ZH-3 had 22 bp and 35 bp deletions. The mutations all led to a frameshift and produced premature stops before the U-box motif and ARM repeats (Figure S1).

### 2.2. Phenotypic Change Due to OsPUB3 Knockout

Phenotypic changes resulting from the knockout of *OsPUB3* were tested in 2021. Three sets of rice materials were used: (a) recipient NIL^ZS97^, T_1_ lines of the two homozygous mutants, and a transgenic-negative control (CK^ZS97^); (b) recipient NIL^MY46^, a T_1_ line of the homozygous mutant, and six T_1_ populations segregating the biallelic mutations; (c) recipient ZH161, six T_2_ homozygous mutants derived from the three biallelic mutants, and a transgenic-negative control (CK^ZH161^). All these populations were measured for four traits of grain shape and size, including grain length (GL), grain width (GW), 1000 grain weight (TGW), and the ratio of grain length to width (RLW). The ZH161-type lines were additionally measured for other five traits, including the number of panicles per plant (NP), number of spikelets per panicle (NSP), number of grains per panicle (NGP), grain yield per plant (GY), and heading date (HD). Student’s *t*-test was performed to analyze phenotypic differences between mutants and controls.

The two homozygous mutants of NIL^ZS97^ both showed significant decreases in GL, GW, and TGW compared with the recipient and CK (Table 1). Decreases in KO-ZS-1 and KO-ZS-2 over CK^ZS97^ were 0.102 and 0.204 mm for GL, 0.080 and 0.122 mm for GW, and 2.14 and 2.67 g for TGW, respectively. The CK itself decreased over the recipient NIL^ZS97^ on these traits. Thus, decreases in the two mutants over NIL^ZS97^ were larger, becoming 0.266 and 0.369 mm for GL, 0.091 and 0.133 mm for GW, and 3.02 and 3.55 g for TGW. Because changes in GL and GW had the same direction, their influences on the GL/GW ratio were mitigated. The two mutants showed non-significant differences with NIL^ZS97^ and significant increases over CK^ZS97^.

The NIL^MY46^ mutants were only tested against the recipient due to unavailable transgenic CK. In each of the six segregating populations, non-significant phenotypic variation was found among the three genotypic groups (data not shown); thus, data of the three genotypes were merged. Together with the homozygous mutant KO-MY-1, a total of seven mutants were analyzed (Table 1). Compared with NIL^MY46^, the mutants all showed decreases in GL, GW, and TGW, which were significant except for GL in KO-MY-1 and KO-MY-3. The decreases in GL, GW, and TGW ranged from 0.010–0.270 mm, 0.065–0.218 mm, and 1.72–4.23 g, and averaged 0.124 mm, 0.143 mm, and 3.02 g, respectively. Compared with the differences between NIL^ZS97^ and its mutants, these effects had the same direction, and the decreases were smaller on GL, larger on GW, and similar on TGW. Accordingly, increases in the GL/GW ratio over the control were much larger in the mutants of NIL^MY46^ than that of NIL^ZS97^.

Among the six homozygous mutants of ZH161, all and five showed significant decreases in GW and TGW over the two controls. Compared with ZH161 and CK^ZH161^, the decreases were averaged as 0.093 and 0.068 mm for GW and 0.052 and 0.072 g for TGW, respectively (Table 1). For GL and RLW, four and six mutants showed significant increases over the two controls. Compared with ZH161 and CK^ZH161^, the increases were averaged as 0.215 and 0.114 mm for GL and 0.184 and 0.111 for RLW, respectively. The mutational directions, compared with that detected in the NIL^ZS97^ and NIL^MY46^ populations, were the opposite for GL but the same for the other three traits. Variations among the three experiments were also found in the magnitude of mutational effects on GW, TGW, and RLW. In accordance with the thinner and smaller grains of ZH161 than NIL^ZS97^ and NIL^MY46^, the knockout caused fewer decreases in GW and TGW when using ZH161 as the recipient, especially in TGW. For RLW, knockout on the *OsPUB3*^MY46^ allele carried by ZH161 and NIL^MY46^ caused much larger increases than on the ZS97 allele carried by NIL^ZS97^. As for the five traits that were only tested in the ZH161 population, the most consistent results were found in the differences in NP, NSP, and NGP between mutants and CK^ZH161^. General decreases in NP and increases in NSP and NGP were observed (Table S1).

In summary, the knockout of *OsPUB3* in the three recipients all resulted in increased RLW and decreased GW and TGW, whereas the effects on GL were less consistent. Meanwhile, the magnitude of the mutational effects on GW, TGW, and RLW varied depending on the target allele as well as the genetic background. NIL^MY46^ is the only recipient on which *OsPUB3* knockout caused large changes for all the RLW, GW, and TGW.

### 2.3. Effects of Expressing OsPUB3 with Rice Actin 1 Promoter

Overexpression transgenic plants were generated to further validate the effects of *OsPUB3*. Since the knockout of *OsPUB3* in NIL^MY46^ caused the largest effect, we amplified the coding sequence of *OsPUB3* from MY46 and introduced it into NIL^MY46^ driven by a rice *Actin 1* promoter. Four independent T_1_ populations, namely OE-1 to OE-4, were selected to measure the four grain-shape traits. The Student’s *t*-test was performed to determine phenotypic differences between negative and positive transgenic plants in each T_1_ population.

Significant differences were detected for all four traits in at least two populations (Table 2). For GL, a significant increase in positive plants over negative plants was observed in each population. The increases ranged from 0.049 to 0.104 mm. For GW, a decrease in positive plants over negative plants was shown in each population but only significant in two populations, OE-1 and OE-4, reduced by 0.039 and 0.042 mm, respectively. For TGW, a significant increase in positive plants over negative plants was detected in two populations, OE-2 and OE-4, rising by 0.84 and 0.46 g, respectively. The other two populations showed a non-significant increase or decrease. For RLW, an increase in positive plants over negative plants was observed in each population, but only significant in three populations, OE-1, OE-3, and OE-4. The significant increases ranged from 0.051 to 0.074. These results showed that overexpression of the *OsPUB3*^MY46^ allele in NIL^MY46^ increased GL but decreased GW. Due to the effect being larger on GL than on GW, increases were observed for RLW and TGW.

## 3. Discussion

Most important agronomic traits are controlled by a few major QTLs and many minor QTLs. Only a small number of genes underlying minor QTLs have been isolated in rice [5,13,23,48,49]. In our previous study, minor QTL *qGS1-35.2* was fine-mapped using NIL populations derived from a cross between ZS97 and MY46. One annotated gene, *OsPUB3*, was found to be the most likely candidate gene. In these NIL populations, the ZS97 allele had a slight effect causing larger GL and smaller GW compared to the MY46 allele, thereby *qGS1-35.2* exhibited a significant effect on RLW [47]. In the present study, the effect of *OsPUB3* was investigated through knockout and overexpression experiments. Knockout of *OsPUB3* in NIL^ZS97^, NIL^MY46^, and ZH161, another cultivar carrying the *OsPUB3*^MY46^ allele, caused consistent effects on RLW, GW, and TGW, as well as significant but less consistent effects on GL. These effects, reflecting differences between function and loss-of-function alleles, are much larger than those detected in the fine-mapping of *qGS1-35.2* that reflected the differences between the functional *OsPUB3*^ZS97^ and *OsPUB3*^MY46^ alleles. Comparing the mutational effects on the four traits between NIL^ZS97^ and NIL^MY46^, changes of 3.28 and 3.02 g in TGW are the closest, and that of 0.000 and 0.098 in RLW are the furthest. This is in agreement with the previous result that the effect of *qGS1-35.2* was significant on grain shape and non-significant on grain weight. These results also confirm our previous hypothesis that the small effect of a minor QTL is a result of the small contrast between partially function alleles carried by the parental lines [5]. There is abundant genetic variation in natural populations, and different alleles of a gene may have different functions [50,51,52]. The identification and screening of different alleles are helpful to rice improvement.

Overexpression and knockout/knockdown of a same gene usually causes opposite effects [10,11,13,15,17,20,23,25,27,28,31,34]. However, we surprisingly discovered that overexpressing and knockout *OsPUB3*^MY46^ both decreased GW. This implied that *OsPUB3* regulated GW by a rare molecular mechanism. *OsPUB3* encodes a U-box type E3 ubiquitin ligase, which has been verified to have ubiquitination activities [53]. There are very few examples of overexpression and knockout/knockdown of the same gene exhibiting a similar phenotype [54,55,56,57,58,59,60,61]. Interestingly, three of them were found to be involved in the ubiquitin-proteasome pathway. *SPL35* regulated rice defense response by interacting with a ubiquitin-conjugating E2 enzyme. Both overexpression and knockdown of *SPL35* caused the lesion to mimic the phenotype [61]. *UNC-45* functioned the organization of myosin. Loss of function of *UNC-45* resulted in paralyzed animals. The overexpressing *UNC-45* promoted the nonnative myosin conformation that was degraded by ubiquitination complexes, displaying a paralysis phenotype as well [55,57,59]. *Usp28* encoded a deubiquitinase that stabilized the subunit of SCF ubiquitin ligase, Fbw7. At the highest level, Usp28 could also stabilize substrates of Fbw7, proto-oncogenes. Knockout of *Usp28* triggered Fbw7 degradation and accumulation of Fbw7 substrates, resulting in oncogenic transformation. Overexpressing *Usp28* stabilized both Fbw7 and its substrates, also causing oncogenic transformation [58]. These examples illustrated that ubiquitination exquisitely regulates protein turnover and homeostasis through a complex interaction system, though the regulatory mechanisms of these genes were different. Whether *OsPUB3* regulated GW through one of the similar molecular mechanisms described above or through other mechanisms remains to be explored.

Our results also showed that the effects of *OsPUB3* varied depending on genetic background. Knockout of *OsPUB3^MY46^* decreased GL in the NIL background but increased the trait in the ZH161 background. As for GW, the magnitude of the mutational effect was much larger in the NIL background than that in the ZH161 background, though knockout *OsPUB3^MY46^* always exhibited decreasing effects. Consequently, the decrease in TGW was approximately five-fold greater in the NIL background than that in the ZH161 background, while the increase in RLW in the NIL background was only half of that in the ZH161 background. The grains of ZH161 were much thinner and smaller compared to those of NIL. The effect variation of *OsPUB3* in the two backgrounds may be related to the QTLs responsible for the difference in grain shape between the two varieties. The genetic interaction between *OsPUB3* and these QTLs would be a good starting point to investigate the molecular mechanisms underlying the role of *OsPUB3* in regulating grain shape and size. Overall, *OsPUB3* regulates grain development through a complex mechanism and is a good target gene for deciphering the regulatory network of grain shape and size in rice.

Grain shape is an important appearance quality, which greatly influences the market value of rice products. Large RLW generally confers better appearance quality and competitive market value. Some breeding programs recently even attempted to convert the traditional short and bold grains of *japonica* rice into long and slender grains because of their excellent appearance quality [55,56]. Due to a more obvious reduction in grain width or increase in grain length, overexpressing *OsPUB3* and knockout in different genetic backgrounds always increased the RLW. Moreover, *OsPUB3* seems to coordinate the trade-off between grain weight and other yield traits. The knockout of *OsPUB3* reduced grain weight but increased the number of panicles or grains, resulting in a stable grain yield. Therefore, *OsPUB3* could be used to improve grain appearance quality without yield penalty through genetically modified breeding.

## 4. Materials and Methods

### 4.1. Knockout OsPUB3 in Three Rice Cultivars

The CRISPR/Cas9 system was used to knockout *OsPUB3*. One target, located at +74 to +93 in the coding region (Figure 1A), was selected using the web-based tool CRISPR-GE (http://skl.scau.edu.cn, accessed on 6 March 2018). The oligonucleotides 3900-cri (Table S2) were designed and ligated into the BGK03 vector (BIOGLE Co., Ltd., Hangzhou, China) according to the manufacturer’s instructions. The original BGK03 vector contains a rice U6 promoter for activating the target sequence, a Cas9 gene driven by the maize ubiquitin promoter, and a hygromycin marker gene driven by the *Cauliflower mosaic virus* 35S promoter.

Three transgenic recipients were used, including NIL^ZS97^, NIL^MY46^, and ZH161. The NIL^ZS97^ and NIL^MY46^ were derived from the cross between ZS97 and MY46, which were previously used for fine-mapping *qGS1-35.2*. ZH161 is an *indica* rice cultivar carrying the *OsPUB3^MY46^* allele. The ORF sequence of *OsPUB3* in ZH161 was analyzed according to the following procedure. Genomic DNA was extracted from a 2 cm long leaf sample of ZH161 using DNeasy Plant Mini Kit (QIAGEN, Hilden, German). Full-length ORF of *OsPUB3* was amplified using the primer pairs 3900-seq (Table S2) and the product was sequenced by the Sanger method.

The CRISPR/Cas9 constructs were separately introduced into three recipients using *Agrobacterium tumefaciens*-mediated transformation. Genomic DNA of T_0_ plants was extracted from a 2 cm long leaf sample using the DNeasy Plant Mini Kit. They were assayed with the hygromycin gene marker Hyg (Table S2). The *OsPUB3* gene fragment was amplified from each Hyg-positive plant using the primer pairs 3900-cri-seq (Table S2). The product was directly sequenced by the Sanger method and decoded using the web-based tool DSDecodeM (http://skl.scau.edu.cn/dsdecode, accessed on 10 May 2019 and 27 July 2020).

### 4.2. Expressing OsPUB3 with Rice Actin 1 Promoter

Total RNA was extracted from leaves of MY46 using RNeasy Plus Mini Kit (QIAGEN, Hilden, German). The 1^st^ strand cDNA was synthesized using ReverTra AceR Kit (Toyobo, Osaka, Japan). The *OsPUB3* cDNA fragment that contained the full coding sequence was amplified using the primer pairs 3900-oe (Table S2). The product was recombined in the pCAMBIA2300 vector using an In-Fusion Advantage Cloning kit (Clontech, Osaka, Japan). The original pCAMBIA2300 vector comprised a rice *Actin1* promoter for activating the target sequence and a neomycin marker gene driven by CaMV 35S promoter. The overexpression construct was introduced into NIL^MY46^ using *Agrobacterium* tumefaciens-mediated transformation. Total DNA was extracted from a 2 cm long leaf sample of transgenic plants using the method of Zheng et al. [62]. Genotypes of transgenic plants were assayed with the neomycin gene marker Neo marker (Table S2).

### 4.3. Field Experiments and Phenotyping

All the rice materials were tested in 2021 at paddy field in the China National Rice Research Institute in Hangzhou, Zhejiang Province, China. Seeds were sown in paddy fields in May and raised in wet bed condition for 26 days. Then, the plants were transplanted with spacing of 16.7 cm between plants and 26.7 cm between rows. In each population, the plants were randomly planted. Normal agricultural practice was employed in field management. From transplanting to harvesting, irrigation was applied to maintain a well-watered condition except that the water was drained for five days at the maximum tillering stage and before harvesting.

For knockout experiment in NIL^ZS97^, each of the four T_1_ homozygous lines contained 10 plants (Table 1). For knockout experiment in NIL^MY46^, 12 plants were planted for the control NIL^MY46^ and 36 T_1_ plants were grown for each of the six segregating populations and one homozygous line. Genotyping was performed using the marker 3900-cri-seq, and those showing an ambiguous genotype were removed. For knockout experiment in ZH161, each of the eight T_2_ homozygous lines contained 30 plants. For overexpression experiment, 30 T_1_ plants were grown for each population. Genotyping was performed using the Neo marker.

In October, plants in each population were individually harvested at maturity for phenotyping. Four traits of grain shape and size, including GL, GW, TGW, and RLW, were measured in all experiments. Approximately 6 g of fully filled grains were divided into two halves and measured for the four traits using an automatic seed counting and analyzing instrument (Model SC-G, Wanshen Ltd., Hangzhou, China). Other five traits, including NP, NSP, NGP, GY, and HD, were additionally measured in the ZH161 knockout experiment. The Student’s *t*-test was performed to determine phenotypic differences between mutants and controls in the knockout experiments and between negative and positive transgenic plants in the overexpression experiment.

## Figures and Tables

**Table 1:** Phenotypic change due to *OsPUB3* knockout.

Population	No. of	Grain Length (mm)			Grain Width (mm)			1000-Grain Weight (g)			Ratio of Grain Length to Width		
	Plants	Mean ± SD	D1 ^a^	D2 ^b^	Mean ± SD	D1	D2	Mean ± SD	D1	D2	Mean ± SD	D1	D2
NILZS97	10	8.387 ± 0.074			3.074 ± 0.036			27.33 ± 0.82			2.738 ± 0.022		
CKZS97	10	8.222 ± 0.068			3.063 ± 0.040			26.45 ± 0.37			2.696 ± 0.040		
KO-ZS-1	10	8.121 ± 0.060	−0.266 **** ^c^	−0.102 **	2.983 ± 0.068	−0.091 ***	−0.080 **	24.31 ± 1.18	−3.02 ****	−2.14 ****	2.737 ± 0.053	−0.001	0.040 *
KO-ZS-2	10	8.018 ± 0.067	−0.369 ****	−0.204 ****	2.941 ± 0.032	−0.133 ****	−0.122 ****	23.78 ± 0.34	−3.55 ****	−2.67 ****	2.738 ± 0.020	0.000	0.042 ****
NILMY46	12	8.417 ± 0.018			3.109 ± 0.008			27.97 ± 0.16			2.715 ± 0.007		
KO-MY-1	36	8.407 ± 0.012	−0.010		2.934 ± 0.007	−0.175 ****		24.57 ± 0.16	−3.40 ****		2.884 ± 0.009	0.169 ****	
KO-MY-2	34	8.367 ± 0.015	−0.050 *		3.014 ± 0.007	−0.095 ****		26.25 ± 0.09	−1.72 ****		2.790 ± 0.008	0.075 ****	
KO-MY-3	35	8.389 ± 0.015	−0.028		2.891 ± 0.015	−0.218 ****		24.03 ± 0.24	−3.94 ****		2.916 ± 0.013	0.201 ****	
KO-MY-4	36	8.314 ± 0.015	−0.103 ****		2.891 ± 0.015	−0.218 ****		23.74 ± 0.22	−4.23 ****		2.895 ± 0.013	0.180 ****	
KO-MY-5	34	8.166 ± 0.017	−0.251 ****		3.044 ± 0.007	−0.065 ****		25.77 ± 0.08	−2.20 ****		2.695 ± 0.008	−0.020	
KO-MY-6	29	8.260 ± 0.018	−0.157 ****		3.004 ± 0.009	−0.105 ****		25.69 ± 0.14	−2.28 ****		2.763 ± 0.009	0.048 ****	
KO-MY-7	35	8.147 ± 0.014	−0.270 ****		2.984 ± 0.006	−0.125 ****		24.59 ± 0.10	−3.38 ****		2.746 ± 0.006	0.031 **	
ZH161	30	7.927 ± 0.091			2.578 ± 0.041			20.24 ± 0.53			3.092 ± 0.040		
CKZH161	30	8.029 ± 0.097			2.552 ± 0.042			20.44 ± 0.35			3.164 ± 0.072		
KO-ZH-1a	30	8.116 ± 0.129	0.189 ****	0.087 **	2.498 ± 0.034	−0.080 ****	−0.054 ****	19.94 ± 0.54	−0.30 *	−0.50 ****	3.271 ± 0.085	0.179 ****	0.106 ****
KO-ZH-1b	30	8.209 ± 0.103	0.282 ****	0.180 ****	2.510 ± 0.041	−0.068 ****	−0.043 ****	20.53 ± 0.61	0.29 *	0.09	3.292 ± 0.056	0.201 ****	0.128 ****
KO-ZH-2a	30	8.049 ± 0.099	0.122 **	0.020	2.495 ± 0.031	−0.082 ****	−0.057 ****	19.90 ± 0.48	−0.34 **	−0.53 ****	3.246 ± 0.055	0.154 ****	0.081 ****
KO-ZH-2b	30	7.904 ± 0.103	−0.023	−0.125 ****	2.475 ± 0.042	−0.103 ****	−0.077 ****	19.32 ± 0.42	−0.92 ****	−1.11 ****	3.213 ± 0.071	0.122 ****	0.049 **
KO-ZH-3a	30	8.089 ± 0.081	0.162 ****	0.060 **	2.457 ± 0.051	−0.120 ****	−0.095 ****	19.58 ± 0.46	−0.66 ****	−0.86 ****	3.314 ± 0.065	0.222 ****	0.149 ****
KO-ZH-3b	30	8.156 ± 0.085	0.229 ****	0.127 ****	2.472 ± 0.037	−0.106 ****	−0.080 ****	19.85 ± 0.36	−0.39 ***	−0.58 ****	3.320 ± 0.049	0.229 ****	0.156 ****

^a^ D1, increase or decrease over the recipient. ^b^ D2, increase or decrease over the transgenic-negative control (CK). ^c^ * *p* < 0.05; ** *p* < 0.01; *** *p* < 0.001; **** *p* < 0.0001.

**Table 2:** Phenotypic performance of *OsPUB3* driven by an *Actin 1* promoter.

Population	Genotype ^a^	No. of	Grain Length (mm)		Grain Width (mm)		1000-Grain Weight (g)		Ratio of Grain Length to Width	
		Plants	Mean ± SD	D ^b^	Mean ± SD	D	Mean ± SD	D	Mean ± SD	D
OE-1	−	11	8.355 ± 0.060		3.124 ± 0.027		27.71 ± 0.49		2.683 ± 0.018	
	+	19	8.404 ± 0.075	0.049 *^c^	3.085 ± 0.046	−0.039 **	27.65 ± 0.59	−0.07	2.733 ± 0.039	0.051 ***
OE-2	−	7	8.271 ± 0.106		3.120 ± 0.045		26.25 ± 0.77		2.661 ± 0.027	
	+	23	8.344 ± 0.076	0.073 *	3.108 ± 0.058	−0.012	27.09 ± 0.63	0.84 **	2.695 ± 0.054	0.033
OE-3	−	5	8.352 ± 0.058		3.081 ± 0.066		26.85 ± 0.91		2.720 ± 0.041	
	+	25	8.435 ± 0.069	0.083 **	3.049 ± 0.057	−0.032	26.99 ± 0.71	0.15	2.777 ± 0.041	0.057 **
OE-4	−	7	8.305 ± 0.055		3.089 ± 0.030		27.03 ± 0.40		2.696 ± 0.037	
	+	22	8.409 ± 0.081	0.104 **	3.047 ± 0.024	−0.042 ***	27.49 ± 0.42	0.46 **	2.770 ± 0.039	0.074 ****

^a^ −, negative transgenic plants; +, positive transgenic plants. ^b^ D, increase or decrease over the negative transgenic plants. ^c^ * *p* < 0.05; ** *p* < 0.01; *** *p* < 0.001; **** *p* < 0.0001.

## Data Availability

Not applicable.

## Supplementary Materials

The following supporting information can be downloaded at: https://www.mdpi.com/article/10.3390/plants11192530/s1, Figure S1: Amino acid sequences of OsPUB3 in the three recipients (NIL^ZS97^, NIL^MY46^, and ZH161) and their homozygous mutants; Table S1: Four yield traits and heading date in ZH161, the transgenic-negative control, and knockout mutants of *OsPUB3*; Table S2: Primers used in this study.
