# Supplementary material for: Control of Grain Shape and Size in Rice by Two Functional Alleles of OsPUB3 in Varied Genetic Background
Source: Plants (Basel). 2022 Sep 27;11(19):2530. doi: 10.3390/plants11192530 (PMC9571118; doi:10.3390/plants11192530)
Supplement: Supplementary file 1 [file plants-11-02530-s001.zip › Figure S1.pdf]

NIL^{ZS97}	MAGNPAAAAPSSSGSSSVFLPPPSPSDGELLRPLHRLARDLSAVDTPAPFLRAAFASISRRSKLLAAAFDDLLLCGAAGELPRSASLCLREVLLVLQRFK	100
NIL^{MY46}	MAGNPAAAAPSSSGSSSVFLPPPSPSDGELLRLLHRLARDLSAVDTPAPFLRAAFASISRRSKLLAAAFDDLLLCGAAGELPRSASLCLREVLLVLQRFK	100
ZH161	MAGNPAAAAPSSSGSSSVFLPPPSPSDGELLRLLHRLARDLSAVDTPAPFLRAAFASISRRSKLLAAAFDDLLLCGAAGELPRSASLCLREVLLVLQRFK	100
KO-ZS-1	MAGNPAAAAPSSSGSSSVFLPPPSPSDGELSGRCTAWRATCPPSTRPRRSCAPRSRPSRGGPSYWRPRSTTCCCAAPRGSCRGRRRCACGRCSWCCSGSR	100
KO-ZS-2	MAGNPAAAAPSSSGSSSVFLPPPSPSDGELSPVAAPPGARPVRRRHARAVPARRVRVHLEAVQATGGRVRRPAAVRRRGGAAAVGVAVPAGGAPGAAAVQ	100
KO-MY-1	MAGNPAAAAPSSSGSSSVFLPPPSPSDGELSPVAAPPGARPVRRRHARAVPARRVRVHLEAVQATGGRVRRPAAVRRRGGAAAVGVAVPAGGAPGAAAVQ	100
KO-MY-2a	MAGNPAAAAPSSSGSSSVFLPPPSPSDGELSPVAAPPGARPVRRRHARAVPARRVRVHLEAVQATGGRVRRPAAVRRRGGAAAVGVAVPAGGAPGAAAVQ	100
Ko-MY-2b	MAGNPAAAAPSSSGSSSVFLPPPSPSDGELSGRCTAWRATCPPSTRPRRSCAPRSRPSRGGPSYWRPRSTTCCCAAPRGSCRGRRRCACGRCSWCCSGSR	100
KO-MY-3a	MAGNPAAAAPSSSGSSSVFLPPPSPSDGELTPVAAPPGARPVRRRHARAVPARRVRVHLEAVQATGGRVRRPAAVRRRGGAAAVGVAVPAGGAPGAAAVQ	100
KO-MY-3b	MAGNPAAAAPSSSGSSSVFLPPPSPSDGELSGRCTAWRATCPPSTRPRRSCAPRSRPSRGGPSYWRPRSTTCCCAAPRGSCRGRRRCACGRCSWCCSGSR	100
KO-MY-4a	MAGNPAAAAPSSSGSSSVFLPPPSPSDGELTPVAAPPGARPVRRRHARAVPARRVRVHLEAVQATGGRVRRPAAVRRRGGAAAVGVAVPAGGAPGAAAVQ	100
KO-MY-4b	MAGNPAAAAPSSSGSSSVFLPPPSPSDGELSGRCTAWRATCPPSTRPRRSCAPRSRPSRGGPSYWRPRSTTCCCAAPRGSCRGRRRCACGRCSWCCSGSR	100
KO-MY-5a	MAGNPAAAAPSSSGSSSVFLPPPSPSDGELPPVAAPPGARPVRRRHARAVPARRVRVHLEAVQATGGRVRRPAAVRRRGGAAAVGVAVPAGGAPGAAAVQ	100
KO-MY-5b	MAGNPAAAAPSSSGSSSVFLPPPSPSDGELSGRCTAWRATCPPSTRPRRSCAPRSRPSRGGPSYWRPRSTTCCCAAPRGSCRGRRRCACGRCSWCCSGSR	100
KO-MY-6a	MAGNPAAAAPSSSGSSSVFLPPPSPSDGELSPVAAPPGARPVRRRHARAVPARRVRVHLEAVQATGGRVRRPAAVRRRGGAAAVGVAVPAGGAPGAAAVQ	100
KO-MY-6b	MAGNPAAAAPSSSGSSSVFLPPPSPSDGELSGRCTAWRATCPPSTRPRRSCAPRSRPSRGGPSYWRPRSTTCCCAAPRGSCRGRRRCACGRCSWCCSGSR	100
KO-MY-7a	MAGNPAAAAPSSSGSSSVFLPPPSPSDGELTPVAAPPGARPVRRRHARAVPARRVRVHLEAVQATGGRVRRPAAVRRRGGAAAVGVAVPAGGAPGAAAVQ	100
KO-MY-7b	MAGNPAAAAPSSSGSSSVFLPPPSPSDGELSGRCTAWRATCPPSTRPRRSCAPRSRPSRGGPSYWRPRSTTCCCAAPRGSCRGRRRCACGRCSWCCSGSR	100
KO-ZH-1a	MAGNPAAAAPSSSGSSSVFLPPPSPSDTAWRATCPPSTRPRRSCAPRSRPSRGGPSYWRPRSTTCCCAAPRGSCRGRRRCACGRCSWCCSGSRRSSPIAR	100
KO-ZH-1b	MAGNPAAAAPSSSGSSSVFLPPPSPGARPVRRRHARAVPARRVRVHLEAVQATGGRVRRPAAVRRRGGAAAVGVAVPAGGAPGAAAVQGDRRRLLGAQPD	100
KO-ZH-2a	MAGNPAAAAPSSSGSSSVFLPPPSPSDGELTPVAAPPGARPVRRRHARAVPARRVRVHLEAVQATGGRVRRPAAVRRRGGAAAVGVAVPAGGAPGAAAVQ	100
KO-ZH-2b	MAGNPAAAAPSSSGSSSVFLPPPSPSDGRCTAWRATCPPSTRPRRSCAPRSRPSRGGPSYWRPRSTTCCCAAPRGSCRGRRRCACGRCSWCCSGSRRSSP	100
KO-ZH-3a	MAGNPAAAAPSSSGSSSVFLPPPSPSDGELSPVAAPPGARPVRRRHARAVPARRVRVHLEAVQATGGRVRRPAAVRRRGGAAAVGVAVPAGGAPGAAAVQ	100
KO-ZH-3b	MAGNPAAAAPSSSGSSSVFLPPPSPSDGELSGRCTAWRATCPPSTRPRRSCAPRSRPSRGGPSYWRPRSTTCCCAAPRGSCRGRRRCACGRCSWCCSGSR	100

NIL^{ZS97}	AIVADCSARSRMRLLLESDEMEAELRELNHDLATLLDLLPVVELGLADDVLDVLALASRQCRRCSPAPESEEALKASVLSLIQEIEREIVPERERLEEIL	200
NIL^{MY46}	AIVADCSARSRMRLLLESDEMEAELRELNHDLATLLDLLPVVELGLADDVLDVLALASRQCRRCSPAPESEEALKASVLSLIQEIEREIVPERERLEEIL	200
ZH161	AIVADCSARSRMRLLLESDEMEAELRELNHDLATLLDLLPVVELGLADDVLDVLALASRQCRRCSPAPESEEALKASVLSLIQEIEREIVPERERLEEIL	200
KO-ZS-1	RSSPIARRAAGCGCCWSPTRWRRSCGSSTTTWPRCSTCCRSSSWGSPTTCSTSSPSRRASAGGARRHRSRRRR.	200
KO-ZS-2	GDRRRLLGAQPDAAAAGVRRDGGGAAGAQPRPGHAARPVAGRRAGARRRRARRPRPRVAPVPAVLAGTGVGGGAEGERAVADTRDRAGDRAGAGEAGGDP	200
KO-MY-1	GDRRRLLGAQPDAAAAGVRRDGGGAAGAQPRPGHAARPVAGRRAGARRRRARRPRPRVAPVPAVLAGTGVGGGAEGERAVADTRDRAGDRAGAGEAGGDP	200
KO-MY-2a	GDRRRLLGAQPDAAAAGVRRDGGGAAGAQPRPGHAARPVAGRRAGARRRRARRPRPRVAPVPAVLAGTGVGGGAEGERAVADTRDRAGDRAGAGEAGGDP	200
KO-MY-2b	RSSPIARRAAGCGCCWSPTRWRRSCGSSTTTWPRCSTCCRSSSWGSPTTCSTSSPSRRASAGGARRHRSRRRR	200
KO-MY-3a	GDRRRLLGAQPDAAAAGVRRDGGGAAGAQPRPGHAARPVAGRRAGARRRRARRPRPRVAPVPAVLAGTGVGGGAEGERAVADTRDRAGDRAGAGEAGGDP	200
KO-MY-3b	RSSPIARRAAGCGCCWSPTRWRRSCGSSTTTWPRCSTCCRSSSWGSPTTCSTSSPSRRASAGGARRHRSRRRR	200
KO-MY-4a	GDRRRLLGAQPDAAAAGVRRDGGGAAGAQPRPGHAARPVAGRRAGARRRRARRPRPRVAPVPAVLAGTGVGGGAEGERAVADTRDRAGDRAGAGEAGGDP	200
KO-MY-4b	RSSPIARRAAGCGCCWSPTRWRRSCGSSTTTWPRCSTCCRSSSWGSPTTCSTSSPSRRASAGGARRHRSRRRR	200
KO-MY-5a	GDRRRLLGAQPDAAAAGVRRDGGGAAGAQPRPGHAARPVAGRRAGARRRRARRPRPRVAPVPAVLAGTGVGGGAEGERAVADTRDRAGDRAGAGEAGGDP	200
KO-MY-5b	RSSPIARRAAGCGCCWSPTRWRRSCGSSTTTWPRCSTCCRSSSWGSPTTCSTSSPSRRASAGGARRHRSRRRR	200
KO-MY-6a	GDRRRLLGAQPDAAAAGVRRDGGGAAGAQPRPGHAARPVAGRRAGARRRRARRPRPRVAPVPAVLAGTGVGGGAEGERAVADTRDRAGDRAGAGEAGGDP	200
KO-MY-6b	RSSPIARRAAGCGCCWSPTRWRRSCGSSTTTWPRCSTCCRSSSWGSPTTCSTSSPSRRASAGGARRHRSRRRR	200
KO-MY-7a	GDRRRLLGAQPDAAAAGVRRDGGGAAGAQPRPGHAARPVAGRRAGARRRRARRPRPRVAPVPAVLAGTGVGGGAEGERAVADTRDRAGDRAGAGEAGGDP	200
KO-MY-7b	RSSPIARRAAGCGCCWSPTRWRRSCGSSTTTWPRCSTCCRSSSWGSPTTCSTSSPSRRASAGGARRHRSRRRR	200
KO-ZH-1a	RAAGCGCCWSPTRWRRSCGSSTTTWPRCSTCCRSSSWGSPTTCSTSSPSRRASAGGARRHRSRRRR	200
KO-ZH-1b	AAAAGVRRDGGGAAGAQPRPGHAARPVAGRRAGARRRRARRPRPRVAPVPAVLAGTGVGGGAEGERAVADTRDRAGDRAGAGEAGGDPGGGRHQRPGELQ	200
KO-ZH-2a	GDRRRLLGAQPDAAAAGVRRDGGGAAGAQPRPGHAARPVAGRRAGARRRRARRPRPRVAPVPAVLAGTGVGGGAEGERAVADTRDRAGDRAGAGEAGGDP	200
KO-ZH-2b	IARRAAGCGCCWSPTRWRRSCGSSTTTWPRCSTCCRSSSWGSPTTCSTSSPSRRASAGGARRHRSRRRR	200
KO-ZH-3a	GDRRRLLGAQPDAAAAGVRRDGGGAAGAQPRPGHAARPVAGRRAGARRRRARRPRPRVAPVPAVLAGTGVGGGAEGERAVADTRDRAGDRAGAGEAGGDP	200
KO-ZH-3b	RSSPIARRAAGCGCCWSPTRWRRSCGSSTTTWPRCSTCCRSSSWGSPTTCSTSSPSRRASAGGARRHRSRRRR	200

NIL^{ZS97}	VEVGINDPASCSEEIESLEQEIGDRASEKNEGAASMIALVGLLRYAKCVLFSATPRPSDSNSKADVEAEDGEPPVPPSDFRCPISLDLMRDPVVVASGQTYD	300
NIL^{MY46}	VEVGINDPASCSEEIESLEQEIGDRASEKNEGAASMIALVGLLRYAKCVLFSATPRPSDSNSKADVEAEDGEPPVPPSDFRCPISLDLMRDPVVVASGQTYD	300
ZH161	VEVGINDPASCSEEIESLEQEIGDRASEKNEGAASMIALVGLLRYAKCVLFSATPRPSDSNSKADVEAEDGEPPVPPSDFRCPISLDLMRDPVVVASGQTYD	300
KO-ZS-1		300
KO-ZS-2	GGGRHQRPGELQRGDREPGAGDRRPCLGEMDGLHDRSRRPPPVCQVRPVQRHASAFRFQFQG	300
KO-MY-1	GGGRHQRPGELQRGDREPGAGDRRPCLGEMDGLHDRSRRPPPVCQVRPVQRHASAFRFQFQG	300
KO-MY-2a	GGGRHQRPGELQRGDREPGAGDRRPCLGEMDGLHDRSRRPPPVCQVRPVQRHASAFRFQFQG	300
KO-MY-2b		300
KO-MY-3a	GGGRHQRPGELQRGDREPGAGDRRPCLGEMDGLHDRSRRPPPVCQVRPVQRHASAFRFQFQG	300
KO-MY-3b		300
KO-MY-4a	GGGRHQRPGELQRGDREPGAGDRRPCLGEMDGLHDRSRRPPPVCQVRPVQRHASAFRFQFQG	300
KO-MY-4b		300
KO-MY-5a	GGGRHQRPGELQRGDREPGAGDRRPCLGEMDGLHDRSRRPPPVCQVRPVQRHASAFRFQFQG	300
KO-MY-5b		300
KO-MY-6a	GGGRHQRPGELQRGDREPGAGDRRPCLGEMDGLHDRSRRPPPVCQVRPVQRHASAFRFQFQG	300
KO-MY-6b		300
KO-MY-7a	GGGRHQRPGELQRGDREPGAGDRRPCLGEMDGLHDRSRRPPPVCQVRPVQRHASAFRFQFQG	300
KO-MY-7b		300
KO-ZH-1a		300
KO-ZH-1b	RGDREPGAGDRRPCLGEMDGLHDRSRRPPPVCQVRPVQRHASAFRFQFQG	300
KO-ZH-2a	GGGRHQRPGELQRGDREPGAGDRRPCLGEMDGLHDRSRRPPPVCQVRPVQRHASAFRFQFQG	300
KO-ZH-2b		300
KO-ZH-3a	GGGRHQRPGELQRGDREPGAGDRRPCLGEMDGLHDRSRRPPPVCQVRPVQRHASAFRFQFQG	300
KO-ZH-3b		300

NIL^{ZS97}	RESIDRWFSSGKSTCPKTGQVLANLELVSNKALKNLISKWCRENGVAMEACEASKSEQAQAVAANKAALEAARMTASFLVKKLSVSFSPDAANRVVHEIR	400
NIL^{MY46}	RESIDRWFSSGKSTCPKTGQVLANLELVSNKALKNLISKWCRENGVAMEACEASKSEQAQAVAANKAALEAARMTASFLVKKLSVSFSPDAANRVVHEIR	400
ZH161	RESIDRWFSSGKSTCPKTGQVLANLELVSNKALKNLISKWCRENGVAMEACEASKSEQAQAVAANKAALEAARMTASFLVKKLSVSFSPDAANRVVHEIR	400
KO-ZS-1		400
KO-ZS-2		400
KO-MY-1		400
KO-MY-2a		400
KO-MY-2b		400
KO-MY-3a		400
KO-MY-3b		400
KO-MY-4a		400
KO-MY-4b		400
KO-MY-5		400
KO-MY-5b		400
KO-MY-6a		400
KO-MY-6b		400
KO-MY-7a		400
KO-MY-7b		400
KO-ZH-1a		400
KO-ZH-1b		400
KO-ZH-2a		400
KO-ZH-2b		400
KO-ZH-3a		400

KO-ZH-3b		400
NIL^{ZS97}	LLSKSGSENRAFVGEAGAVPLLVPLLYSEDAGLQLNAVTALLNLSILEANKKRIMHADGAVEAVAHIMSSGATWRAKENAAAAVLSLASVHSYRRRLGRN	500
NIL^{MY46}	LLSKSGSENRAFVGEAGAVPLLVPLLYSEDAGLQLNAVTALLNLSILEANKKRIMHADGAVEAVAHIMSSGATWRAKENAAAAVLSLASVHSYRRRLGRN	500
ZH161	LLSKSGSENRAFVGEAGAVPLLVPLLYSEDAGLQLNAVTALLNLSILEANKKRIMHADGAVEAVAHIMSSGATWRAKENAAAAVLSLASVHSYRRRLGRN	500
KO-ZS-1		500
KO-ZS-2		500
KO-MY-1		500
KO-MY-2a		500
KO-MY-2b		500
KO-MY-3a		500
KO-MY-3b		500
KO-MY-4a		500
KO-MY-4b		500
KO-MY-5a		500
KO-MY-5b		500
KO-MY-6a		500
KO-MY-6b		500
KO-MY-7b		500
KO-MY-7b		500
KO-ZH-1a		500
KO-ZH-1b		500
KO-ZH-2a		500
KO-ZH-2b		500
KO-ZH-3a		500
KO-ZH-3b		500
NIL^{ZS97}	QSVVEKLVHLVRTGPTSTKKDALAALLTLAGERENVGKLVDAGVAEVALSAISKEETAAAVLAALAKRGGAEAIVNIDGAVARLVAEMRRGTDWARENAT	600
NIL^{MY46}	QSVVEKLVHLVRTGPSSTKKDALAALLTLAGERENVGKLVDAGVAEVALSAISKEETAAAVLAALAKRGGAEAIVNIDGAVARLVAEMRRGTDWARENAT	600
ZH161	QSVVEKLVHLVRTGPSSTKKDALAALLTLAGERENVGKLVDAGVAEVALSAISKEETAAAVLAALAKRGGAEAIVNIDGAVARLVAEMRRGTDWARENAT	600
KO-ZS-1		600
KO-ZS-2		600
KO-MY-1		600
KO-MY-2a		600
KO-MY-2b		600
KO-MY-3a		600
KO-MY-3b		600
KO-MY-4a		600
KO-MY-4b		600
KO-MY-5a		600
KO-MY-5b		600
KO-MY-6a		600
KO-MY-6b		600
KO-MY-7a		600
KO-MY-7b		600
KO-ZH-1a		600
KO-ZH-1b		600
KO-ZH-2a		600
KO-ZH-2b		600
KO-ZH-3a		600
KO-ZH-3b		600
NIL^{ZS97}	AALVLLCRRLGAPAVTQVMAVPGVEWAIWELMSIGTERARRKAASLGRICRRWAAASAADGERGGGCPVATVVPPAMMAS	680
NIL^{MY46}	AALVLLCRRLGAPAVTQVMAVPGVEWAIWELMSIGTERARRKAASLGRICRRWAAASAADGERGGGCPVATVVPPAMMAS	680
ZH161	AALVLLCRRLGAPAVTQVMAVPGVEWAIWELMSIGTERARRKAASLGRICRRWAAASAADGERGGGCPVATVVPPAMMAS	680
KO-ZS-1		680
KO-ZS-2		680
KO-MY-1		680
KO-MY-2a		680
KO-MY-2b		680
KO-MY-3a		680
KO-MY-3b		680
KO-MY-4a		680
KO-MY-4b		680
KO-MY-5a		680
KO-MY-5b		680
KO-MY-6a		680
KO-MY-6b		680
KO-MY-7a		680
KO-MY-7b		680
KO-ZH-1a		680
KO-ZH-1b		680
KO-ZH-2a		680
KO-ZH-2b		680
KO-ZH-3a		680
KO-ZH-3b		680

Figure S1. Amino acid sequences of OsPUB3 in the three recipients (NIL^{ZS97}, NIL^{MY46} and ZH161) and their homozygous mutants. The U-box motif and Armadillo repeat are highlighted in blue and green, respectively. Differences among the three recipients are highlighted in yellow. Differences between the mutants and the recipients are indicated in red letters.
